# Salidroside mitigates cognitive deficits in AlCl_3_ exposed aging mouse by modulating APP processing and mitochondrial dysfunction

**DOI:** 10.3389/fnbeh.2026.1802923

**Published:** 2026-05-05

**Authors:** Yiru Dong, Huiling Jin, Shengmin Wang, Yanji Xu

**Affiliations:** Neurotoxicology Laboratory, Department of Preventive Medicine, Yanbian University, Yanji, Jilin, China

**Keywords:** Alzheimer’s disease, amyloid-beta, APP processing, mitochondrial dysfunction, salidroside

## Abstract

**Background and objectives:**

Mitochondrial dysfunction and oxidative stress are key contributors to the progression of Alzheimer’s disease (AD). Salidroside, a bioactive glycoside derived from *Rhodiola rosea*, exhibits neuroprotective and antioxidative properties; however, its effects on mitochondrial dysfunction and APP processing in AD remain to be fully elucidated.

**Methods and study design:**

We employed both *in vivo* and *in vitro* models to evaluate the neuroprotective potential of salidroside. D-galactose-induced AlCl3 exposed aging mouse model was used for behavioral assessments, biochemical analyses of brain tissue biomarkers, and evaluation of mitochondrial dysfunction-related proteins and functions. *In vitro* experiments with HT-22 hippocampal neurons assessed the effects of salidroside on oxidative stress, mitochondrial integrity, apoptosis, and amyloid precursor protein (APP) processing.

**Results:**

Salidroside significantly improved cognitive performance and reduced Aβ deposition in the AlCl3 exposed aging mouse by modulating APP processing, characterized by downregulation of β- and γ-secretase activities and enhancement of α-secretase activity. These changes coincided with decreased mitochondrial protein aggregation and restored mitochondrial function and redox balance. *In vitro*, salidroside attenuated reactive oxygen species (ROS) generation, inhibited neuronal apoptosis, and suppressed Aβ production, demonstrating broad neuroprotective effects relevant to AD pathology.

**Conclusion:**

Our results suggest that salidroside may alleviate mitochondrial dysfunction and reduce mitochondrial protein aggregation by modulating APP processing, promoting sAPPα production while decreasing β-CTF and Aβ levels. These findings provide preliminary evidence supporting the neuroprotective potential of salidroside in ameliorating mitochondrial impairment and cognitive deficits associated with Alzheimer’s disease, warranting further investigation.

## Highlights

- Salidroside enhances learning and memory abilities in mouse model of AlCl3 exposed aging mouse.
- Salidroside modulates APP cleavage by enhancing α-secretase and inhibiting β- and γ-secretase activities.
- Salidroside potentially reduces mitochondrial protein aggregation and improves mitochondrial function.
- Salidroside may regulate sphingolipid metabolism as a new observational marker in neurodegeneration.
- These effects suggest a multi-target neuroprotective role of salidroside as a natural supplement.

## Introduction

Alzheimer’s disease is a progressive neurodegenerative disorder and the leading cause of dementia (Ballard et al., 2011). Global estimates from the World Alzheimer Report indicate that approximately 55 million individuals were living with dementia in 2020, a number projected to nearly triple to over 150 million by 2050 (Knopman et al., 2021). Furthermore, when using a biological definition of AD rather than solely clinical criteria, prevalence rates are estimated to be threefold higher, highlighting a large population with pre-clinical pathology (Tahami Monfared et al., 2022; Iso-Markku et al., 2022). Clinically, AD typically begins with subtle memory decline and progressively leads to language impairment, executive dysfunction, and loss of autonomy (Iso-Markku et al., 2022; Jessen et al., 2020; Teunissen et al., 2022).

Despite decades of research, no disease-modifying therapy has been successful. Current FDA-approved treatments, including cholinesterase inhibitors and memantine, offer only symptomatic relief without halting underlying neurodegeneration (Breijyeh and Karaman, 2020; Cummings, 2021). These stark realities underscore the urgent need for novel interventions that target multiple pathways and may alter the course of the disease.

A hallmark of AD pathophysiology is the aberrant proteolytic processing of APP, which leads to the accumulation of amyloid-β (Aβ) peptides, particularly Aβ1-42, through the amyloidogenic cascade mediated by β- and γ-secretases (Bera et al., 2020; Ma et al., 2022; Zyśk et al., 2023). In contrast, non-amyloidogenic cleavage by α-secretase precludes Aβ formation and releases the neurotrophic fragment sAPPα (Al-Kuraishy et al., 2023; Zhang et al., 2021). Accumulating evidence indicates that sAPPα not only exerts trophic effects but also directly inhibits BACE1 (β-secretase), thereby reducing Aβ generation (Nithianandam et al., 2023; Jorfi et al., 2023). The pathological shift toward amyloidogenic APP processing triggers synaptic dysfunction, oxidative stress, mitochondrial compromise, and neuroinflammation, underscoring the therapeutic appeal of strategies that simultaneously repress Aβ formation and enhance sAPPα production (Kim et al., 2021).

However, clinical trials targeting β- or γ-secretases have largely failed due to off-target toxicity, poor CNS penetration, or minimal impact at late disease stages (Wang H. et al., 2020). Against this backdrop, naturally derived compounds and traditional herbal medicines have gained traction as multi-targeted, potentially safer alternatives (Zhang N. et al., 2023). One such candidate, salidroside, a phenylethanoid glycoside derived from *Rhodiola rosea*, has demonstrated antioxidative, anti-inflammatory, anti-apoptotic, and mitochondrial stabilizing effects in preclinical neurological models (Yang et al., 2022; Yao et al., 2022). Nevertheless, its influence on APP processing and Aβ dynamics in AD contexts remains underexplored.

In this study, we employed a network pharmacology-guided integrative approach encompassing *in vivo* AD mouse models and *in vitro* hippocampal neuron assays to investigate the mechanistic actions of salidroside. Utilizing a D-galactose plus AlCl3-induced mouse model, we assessed spatial learning and memory via validated behavioral paradigms. Bioinformatic target prediction revealed salidroside’s intersection with AD-related pathways, notably those governing APP processing and mitochondrial integrity. Subsequent biochemical assays confirmed that salidroside attenuates BACE1 expression and activity, shifting APP processing toward the non-amyloidogenic route and amplifying sAPPα levels (Yao et al., 2022). Concurrently, salidroside preserved mitochondrial function, bolstered antioxidant enzyme activity, and attenuated neuronal apoptosis in both mouse hippocampal tissue and HT22 neuronal cultures. Collectively, our findings furnish robust experimental support for salidroside as a multi-functional neuroprotective agent that modulates APP processing, safeguards mitochondrial homeostasis and mitigates neuronal loss, offering a mechanistic foundation for its further development as an AD therapeutic candidate.

## Materials and methods

### Animal model and drug intervention protocols

To investigate the neuroprotective effects of salidroside on cognitive impairment associated with Alzheimer’s pathology, a D-galactose and aluminum chloride-induced aging model was established in male Kunming (KM) mouse. Salidroside, a primary bioactive compound isolated from *Rhodiola rosea*, is known for its anti-inflammatory, antioxidant, and anti-aging properties. In this study, the compound was derived from a laboratory-prepared batch previously used in related research (project code: SCI10661042200511) and freshly dissolved in double-distilled water at concentrations of 25, 50, and 100 mg/kg/day. Fifty KM mouse (2 months old, 40 ± 2 g) were randomly divided into five groups (*n* = 10 per group): Control (CT), Model (AE), and three salidroside-treated groups receiving low (Low-Sal), medium (Med-Sal), and high (High-Sal) doses. The mouse were housed in a SPF environment (22 °C–25 °C, 35%–60% humidity, 12-h light/dark cycle) at the Animal Center of Yanbian University. From day 1 to day 50, the AE and salidroside groups received daily intraperitoneal injections of D-galactose (120 mg/kg) in the morning and AlCl3 (20 mg/kg) in the evening to induce neurodegenerative changes. Mouse in the salidroside groups additionally received oral administration of the compound at the indicated doses once daily at noon. Control mouse received equivalent volumes of normal saline. Injection sites were alternated between the left and right abdominal cavities to minimize tissue damage. All animals were subjected to behavioral and biochemical assessments at the end of the 50-days intervention (Table 1).

**TABLE 1:** Experimental grouping and exposure dose (*n* = 10).

No.	Group	Drug	Dose/day	Mode
1	CT (control)	0.9% NaCl	120 mg/kg	IP
		0.9 NaCl	120 mg/kg	IG
2	AE (model)	D-Gal	120 mg/kg	IP
		AlCl_3_	20 mg/kg	IP
		0.9 NaCl	100 mg/kg	IG
3	Low-salidroside	D-Gal	120 mg/kg	IP
		AlCl_3_	20 mg/kg	IP
		Salidroside	25 mg/kg	IP
4	Medium-salidroside	D-Gal	120 mg/kg	IP
		AlCl_3_	20 mg/kg	IP
		Salidroside	50 mg/kg	IG
5	High-salidroside	D-Gal	120 mg/kg	IP
		AlCl_3_	20 mg/kg	IP
		Salidroside	100 mg/kg	IG

Experimental grouping and dose, the experimental animals were randomly divided into five groups, and the three drugs were administered indifferent ways in the early, middle and late periods.

### Behavioral assessments of cognitive function

To assess cognitive outcomes following salidroside treatment, a series of behavioral tests targeting spatial learning, fear-associated memory, and short-term retention were conducted during the final intervention phase (days 42–50). These included the Morris water maze, passive avoidance, and step-down tests. In the Morris water maze (days 42–46), mouse underwent 4 days of acquisition training to locate a hidden platform in a circular pool, followed by a probe trial to evaluate spatial memory, measured by time in the target quadrant and platform crossings. Fear memory was assessed using the passive avoidance task, wherein step-through latency and error frequency were recorded 24 h after a conditioned foot shock. The step-down test evaluated short-term memory based on latency to descend from a safe platform onto an electrified grid and the number of avoidance failures. Behavioral performances were recorded using automated video tracking systems under standardized light-phase conditions.

### Network pharmacology analysis and target prediction

To elucidate the potential therapeutic mechanisms of salidroside in AD, we first retrieved salidroside-related targets from the Traditional Chinese Medicine Systems Pharmacology (TCMSP) database and GeneCards, and compiled AD-associated genes from GeneCards and the Online Mendelian Inheritance in Man (OMIM) database. These datasets were standardized and cross-referenced using UniProt identifiers to ensure consistency. We identified overlapping genes between salidroside-related targets and AD-associated genes via Venn diagram analysis, considering these common targets as candidate molecules for further investigation. Subsequently, a protein-protein interaction (PPI) network was constructed based on the STRING database with a confidence score threshold set at 0.4. This network was imported into Cytoscape software for topological analysis, where key hub genes were identified using centrality measures such as degree, betweenness, and closeness calculated by the CytoNCA and CentiScaPe plug-ins. To elucidate the biological functions and pathways these targets may influence, Gene Ontology (GO) and Kyoto Encyclopedia of Genes and Genomes (KEGG) enrichment analyses were performed using DAVID Bioinformatics Resources. Enriched terms with statistical significance (adjusted *p*-value < 0.05) informed the selection of relevant signaling pathways and biological processes, providing a theoretical foundation for subsequent experimental validation.

### Brain tissue sampling and mitochondrial isolation

Following completion of behavioral assessments, mouse were euthanized, and their brains were rapidly excised and weighed. For mitochondrial isolation, brain tissues were mechanically homogenized using an ultrasonic cell disruptor in ice-cold mitochondrial isolation buffer A, following the manufacturer’s protocol (Tissue Mitochondria Isolation Kit). The homogenate was subjected to sequential centrifugation steps at 600 × *g* for 5 min to remove cell debris and nuclei, followed by 11,000 × *g* for 10 min to pellet mitochondria. The mitochondrial pellet was collected and lysed for downstream biochemical assays. In parallel, whole brain tissue lysates were prepared by finely chopping brain samples, followed by incubation in RIPA lysis buffer at a ratio of 20 mg tissue per 200 μL buffer. Samples were sonicated thoroughly to ensure complete cell disruption, then centrifuged at 14,000 × *g* for 3 min at 4 °C to remove insoluble debris. The resulting supernatant was collected for protein quantification and further analysis. Isolated mitochondria and brain lysates served as sources for detecting key biochemical markers. Mitochondrial function was assessed by measuring the activities of respiratory chain complexes I and III, cytochrome c (Cyt-C), and ATPase using commercially available ELISA kits. Meanwhile, APP processing intermediates, including β-C-terminal fragment (β-CTF) and Aβ_1–42_ peptide, were quantified by ELISA. Additionally, levels of secretase enzymes α-, β-, and γ-secretase, soluble APPα (sAPPα), and APP N-terminal fragments were measured in brain lysates to evaluate APP cleavage dynamics.

### Detection of APP processing pathway in mouse brain

Key enzymes and cleavage products involved in the APP proteolytic pathway were quantified using ELISA to investigate the modulation of APP processing *in vivo*. Brain tissues were harvested post-intervention, and whole brain lysates were prepared by homogenizing tissue fragments in RIPA lysis buffer (200 μL per 20 mg tissue), followed by ultrasonication and centrifugation at 14,000 × *g* for 3 min at 4 °C. The resulting supernatants were collected for downstream analysis. ELISA assays were performed according to manufacturers’ protocols to quantify the concentrations of α-secretase, β-secretase, and γ-secretase, as well as their downstream products, including sAPPα, β-C-terminal fragment (β-CTF), and amyloid β peptide (Aβ_1–42_). Commercially available ELISA kits (KQBioscience, China) were used for all quantitative measurements. Protein concentrations were normalized to total protein content assessed by bicinchoninic acid (BCA) assay.

In addition, Western blotting was employed exclusively to validate the expression level of BACE1 protein in brain lysates. Equal amounts of protein were resolved by SDS-PAGE and transferred onto PVDF membranes. The membranes were incubated with anti-BACE1 primary antibody followed by appropriate HRP-conjugated secondary antibody. Protein bands were visualized using chemiluminescent detection and quantified via densitometry analysis. These assays provided a focused evaluation of BACE1 expression within the APP cleavage context under different treatment conditions.

### Evaluation of mitochondrial function and oxidative status

To assess mitochondrial functional integrity and redox homeostasis in the mouse brain, a series of biochemical assays were conducted on both mitochondrial-enriched fractions and whole-brain homogenates. Mitochondria were isolated using a commercially available tissue mitochondria isolation kit (KQBioscience, China) according to the manufacturer’s protocol. Briefly, following behavioral testing, fresh brain tissue was weighed and homogenized in ice-cold mitochondrial isolation buffer using an ultrasonic disruptor. The homogenate was subjected to sequential centrifugation at 600 × *g* for 5 min and 11,000 × *g* for 10 min to obtain a mitochondrial pellet, which was lysed for subsequent analysis. The mitochondrial functional status was evaluated by quantifying ATP synthase (ATPase) activity, mitochondrial respiratory chain complex I and III activities, and the levels of cytochrome c (Cyt-C), using ELISA kits following the manufacturers’ instructions. Additionally, sphingomyelin (SM), a lipid component associated with mitochondrial membrane stability, was also measured. Oxidative stress parameters were assessed in both cytoplasmic and mitochondrial fractions. Reactive oxygen species (ROS) levels were measured using dichlorodihydrofluorescein diacetate (DCFH-DA) staining, while mitochondrial-specific ROS (mtROS) were quantified using MitoSOX Red reagent. After staining, fluorescence intensity was determined by flow cytometry (Beckman Coulter), with at least 10,000 cells acquired per sample. Moreover, endogenous antioxidant capacity was evaluated by measuring the enzymatic activities or levels of catalase (CAT), glutathione (GSH), and malondialdehyde (MDA), all of which are widely recognized indicators of redox status. All biochemical indices were normalized to total protein content, and data were interpreted in relation to treatment group comparisons. These measures collectively provided a multi-dimensional evaluation of mitochondrial energetics and oxidative damage under neurodegenerative conditions.

### HT-22 cell culture and functional validation *in vitro*

The murine hippocampal neuronal cell line HT-22 was employed to explore the *in vitro* effects of salidroside on oxidative stress, mitochondrial integrity, and apoptosis. Cells were cultured in DMEM supplemented with 10% fetal bovine serum and maintained at 37 °C in a humidified incubator with 5% CO2 (Table 2). For viability assays, cells were seeded into 96-well plates at a density of 1.5 × 104 cells/well. After 24 h of adherence, cells were exposed to different concentrations of salidroside or control treatments. Cell viability was assessed 8 h post-treatment using the Cell Counting Kit-8 (CCK-8; Dojindo, Japan), and absorbance was measured at 450 nm using a microplate reader.

**TABLE 2:** *In vitro* experimental grouping.

Group	Processing method
Con	The same volume of DMEM complete medium 80 nmol/L OA (aq)
OA	80 nmol/L OA (aq)
OA+Sal(L)	80 nmol/L OA + 100 μmolL Sal (aq)
OA+Sal(M)	80 nmol/L OA + 200 μmolL Sal (aq)
OA+Sal(H)	80 nmol/L OA + 300 μmolL Sal (aq)
OA+DP	80 nmol/L OA + 50 μmolL DP (aq)

To assess apoptosis, HT-22 cells were seeded in 6-well plates (2.0 × 105 cells/well), treated with experimental agents, and stained using Annexin V-FITC and propidium iodide (PI) (Table 3). Cells were harvested with trypsin-EDTA-free solution, washed, resuspended in binding buffer, and incubated with fluorescent probes for 15 min in the dark at room temperature. Stained cells were analyzed by flow cytometry (FACSCalibur, BD Biosciences) to determine the percentage of early and late apoptotic cells. ROS and mtROS levels were evaluated using DCFH-DA and MitoSOX Red, respectively. Briefly, after drug treatment, cells were incubated with 10 μmol/L DCFH-DA or 5 μmol/L MitoSOX working solutions for 30 min at 37 °C in the dark. After thorough washing with PBS, fluorescence intensity was detected via flow cytometry under excitation/emission wavelengths of 488/525 nm. At least 10,000 events per sample were collected, and ROS levels were expressed as median fluorescence intensity normalized to control.

**TABLE 3:** Apoptosis was detected by Annexin V-FITC/PI double staining.

Cell state	Normal	Early apoptosis	Late apoptosis	Necrosis
Annexin V-FITC	−	+	+	−
PI	−	−	+	+
Quadrant	Q4	Q3	Q2	Q1

For mitochondrial functional assays, cells were subjected to differential centrifugation following lysis in a mitochondrial isolation buffer supplemented with PMSF. Mitochondria were collected by centrifugation at 11,000 × *g*, lysed, and used for downstream analyses including measurement of ATPase activity and mitochondrial complexes I and III using ELISA kits. Oxidative status was further evaluated by quantifying intracellular antioxidant enzyme activities, superoxide dismutase (SOD), catalase (CAT), glutathione (GSH), and lipid peroxidation marker malondialdehyde (MDA), according to manufacturer protocols. Collectively, these assays provided a comprehensive readout of salidroside’s cellular effects, confirming its neuroprotective potential through mitochondrial stabilization, oxidative stress attenuation, and anti-apoptotic activity *in vitro*.

### Immunofluorescence staining and co-localization analysis

HT-22 cells were seeded on glass-bottom dishes and divided into five groups: control (CT), Aβ1-42, and Aβ1-42 combined with Sal at low, medium, and high concentrations. After treatments, the cells were washed three times with phosphate-buffered saline (PBS) and incubated with 100 nM MitoTracker CMXRos Red at 37 °C for 30 min to label mitochondria. Cells were then fixed with 4% paraformaldehyde for 15 min at room temperature, followed by permeabilization with 0.1% Triton X-100 for 10 min.

Blocking was performed for 1 h at room temperature using 5% bovine serum albumin in PBS. Subsequently, cells were incubated overnight at 4 °C with a primary antibody against the C-terminal fragment (CTF) at a dilution of 1:200. After washing with PBS, the cells were incubated with a FITC-conjugated secondary antibody for 1 h at room temperature in the dark. Nuclear staining was performed with DAPI (1 μg/mL) for 5 min.

Following washing and mounting with an antifade medium, fluorescence images were acquired using a fluorescence microscope equipped with appropriate filters for DAPI (blue), FITC (green), and MitoTracker (red). Co-localization of mitochondria and CTF signals was quantified using image analysis software.

### Statistical analysis

All experimental data are presented as mean ± standard deviation (SD). Statistical analyses were performed using SPSS software version 27.0 (IBM, USA). For comparisons among multiple groups, one-way analysis of variance (ANOVA) was employed, followed by Tukey’s *post-hoc* test to assess pairwise differences. For behavioral data collected over time (e.g., water maze learning curves), repeated-measures ANOVA was applied. Statistical significance was defined as *P* < 0.05. All experiments were conducted in at least three independent biological replicates unless otherwise specified. Graphs were generated using GraphPad Prism version 9.0 (GraphPad Software, USA). The researchers conducting behavioral scoring and data quantification were blinded to group allocation to minimize bias.

## Results

### Effects of salidroside on behavioral changes and acetylcholinesterase activity in AlCl3 exposed aging mouse

The combined intoxication of aluminum and D-galactose has been extensively utilized as a well-validated animal model that replicates key features of aging disease-related neurodegeneration, particularly cognitive decline and memory dysfunction. In this study, we systematically evaluated the protective effects of salidroside–a natural bioactive compound–on cognitive deficits induced by this neurotoxic challenge. Multiple behavioral assessments were employed to comprehensively assess different cognitive domains, including associative learning, short-term memory, and spatial navigation.

**FIGURE 1:**
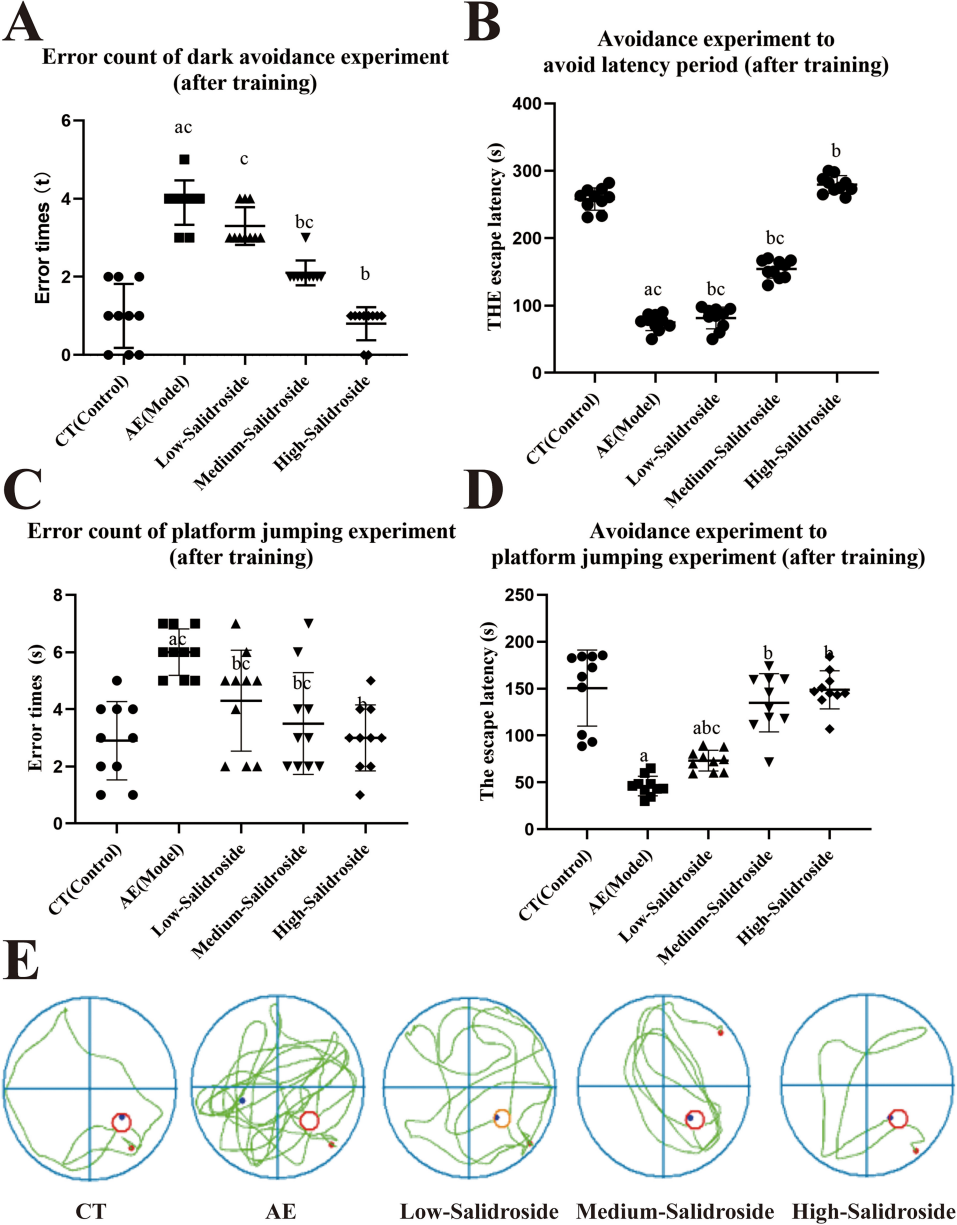
Behavioral performance in AlCl3 exposed aging mouse and effects of salidroside treatment at low, medium, and high doses. **(A)** Step-through errors were elevated in AE mouse but decreased gradually following salidroside treatment, nearly normalized at the high dose. **(B)** Step-through latency was reduced in AE mouse and significantly prolonged after salidroside administration, most notably in the high-dose group. **(C)** Step-down errors were significantly increased in AE mouse compared to controls (Con), whereas salidroside dose-dependently reduced error counts. **(D)** Step-down latency was shortened in AE mouse but progressively restored toward control levels by salidroside treatment, with the high-dose group showing the greatest improvement. **(E)** Representative water maze path maps illustrating dose-dependent improvement in spatial navigation with salidroside, transitioning from disordered routes in AE mouse to ordered paths approaching control (CT) mouse. Data are presented as mean ± SD (*n* = 10). Statistical analysis was conducted using one-way ANOVA followed by Tukey’s *post-hoc* test. a:*P* < 0.05 vs. control; b:*P* < 0.05 vs. AE group; c:*P* < 0.05 vs. high-salidroside group.

In the passive avoidance test, mouse subjected to aluminum and D-galactose intoxication (AE group) demonstrated marked impairments in fear-associated memory, as reflected by a significantly increased number (*F*_(4, 45)_ = 62.57; *P* < 0.0001) of errors and a shortened latency (*F*_(4, 45)_ = 444.1; *P* < 0.0001) to enter the dark chamber compared with control animals. These results indicate deficits in memory retention and consolidation processes. Administration of salidroside resulted in significant dose-dependent improvements. Notably, the high-dose group (100 mg/kg) displayed latencies and error rates closely approximating those seen in controls, indicating substantial recovery of memory function (Figures 1A,B).

The step-down test, implemented to evaluate short-term memory, revealed that AE mouse exhibited reduced latency (*F*_(4, 45)_ = 35.45; *P* < 0.0001) to step down and increased error counts (*F*_(4, 45)_ = 8.031; *P* = 0.0001), signifying impairment in immediate memory processing. Salidroside treatment ameliorated these deficits in a progressive, dose-dependent manner, with medium- and high-dose groups showing statistically significant improvements in latency (*F*_(4, 45)_ = 35.45; *P* < 0.0001) and error reduction (*F*_(4, 45)_ = 8.031; *P* = 0.0002) compared to AE mouse (Figures 1C,D).

Spatial learning and memory were further assessed using the Morris water maze, considered a gold standard for hippocampus-dependent cognitive evaluation. AE mouse demonstrated disorganized and inefficient swimming paths, as well as a decrease in time spent in the target quadrant during probe trials, indicative of spatial memory impairment. Conversely, salidroside-treated mouse exhibited marked improvements in their search strategies and preferential occupancy of the target quadrant (*F*_(4, 45)_ = 1.600; *P* = 0.029), also in a dose-responsive fashion. High-dose salidroside administration almost restored spatial navigation performance to control levels, as evidenced by representative swim trajectories (Figure 1E and Tables 4, 5).

**TABLE 4:** Evasion incubation period of positioning navigation experiment ($\bar{x}$ s *n* = 10).

Group	1st day	2nd day	3rd day	4th day
CT (control)	78.57 ± 26.38	100.06 ± 19.29	70.88 ± 26.52	61.72 ± 22.20
AE (model)	112.81 ± 6.63^a^	110.53 ± 10.84^c^	105.74 ± 15.82^ac^	110.57 ± 9.57^ac^
Low-salidroside	104.06 ± 14.68^a^	99.44 ± 23.43	91.46 ± 18.66^a^	95.44 ± 10.92^abc^
Medium-salidroside	102.37 ± 14.68^a^	93.40 ± 16.71^b^	86.38 ± 9.52b	79.54 ± 12.38^abc^
High-salidroside	99.34 ± 19.16^a^	91.53 ± 11.89^b^	85.27 ± 15.95^b^	63.92 ± 19.63^b^

Results of the evasion incubation period in the positioning navigation test. Data met normality and homogeneity assumptions. One-way ANOVA showed significant differences between the model and control groups, and between treatment and model groups. (^a^*p* < 0.05) vs. control; (^b^*p* < 0.05) vs. model; (*^c^p* < 0.05) vs. high-salidroside.

**TABLE 5:** Space exploration experiment mouse looking for platform experiment results ($\bar{x}$± s *n* = 10).

Group	Target quadrant dwell time (s)	The number of times the platform has been crossed (t)	Swimming speed (mm/s)
CT (control)	37.95 ± 15.76	8.10 ± 5.15	170.85 ± 6.42
AE (model)	25.81 ± 12.45^c^	4.40 ± 3.16^ac^	170.62 ± 13.54
Low-salidroside	32.77 ± 10.37	5.22 ± 1.20^abc^	170.93 ± 2.95
Medium-salidroside	38.27 ± 18.47	6.00 ± 4.57^ac^	170.70 ± 5.67
High-salidroside	40.82 ± 15.61^b^	8.20 ± 1.98^ab^	170.56 ± 13.36

Results of the spatial navigation (platform search) experiment. Data met normality and homogeneity assumptions. One-way ANOVA showed significant differences between the model and control groups, and between treatment and model groups. (^a^*p* < 0.05) vs. control; (^b^*p* < 0.05) vs. model; (*^c^p* < 0.05) vs. high-salidroside.

To elucidate the neurochemical substrates underlying these behavioral alterations, acetylcholinesterase (AChE) activity was measured in the hippocampus and cortex using a commercially available kit provided by Nanjing Jiancheng Bioengineering Institute. This enzymatic assay revealed that aluminum and D-galactose intoxication significantly elevated AChE activity in both brain regions, indicative of enhanced acetylcholine hydrolysis and consequent cholinergic dysfunction, which is consistent with the observed cognitive deficits. Importantly, salidroside administration dose-dependently attenuated AChE activity (*F*_(4, 20)_ = 6.382; *P* = 0.0043), suggesting a restoration of cholinergic neurotransmitter balance and providing a plausible biochemical mechanism underlying the cognitive improvements observed (Table 6).

**TABLE 6:** Effect of SAL on neurotransmitter - acetylcholinesterase activity ($\bar{x}$± s).

Group	ACHE (U/mg)
CT (control)	1.31 ± 0.13
AE (model)	2.40 ± 0.32^ac^
Low-salidroside	2.01 ± 0.67
Medium-salidroside	1.79 ± 0.46
High-salidroside	1.33 ± 0.28^b^

Results of acetylcholinesterase (AChE) activity assay. Data met normality and homogeneity assumptions. One-way ANOVA showed significant differences between the model and control groups, and between treatment and model groups. (^a^*p* < 0.05) vs. control; (^b^*p* < 0.05) vs. model; (^c^*p* < 0.05) vs. high-salidroside.

Salidroside effectively mitigates the cognitive impairments induced by combined aluminum and D-galactose intoxication, across multiple behavioral paradigms encompassing associative learning, short-term memory, and spatial navigation. This cognitive improvement is paralleled by a normalization of AChE activity in key brain regions. Collectively, these findings offer compelling *in vivo* evidence of salidroside’s neuroprotective and cognitive-enhancing effects, underscoring its potential as a therapeutic candidate for neurodegenerative disorders characterized by cholinergic dysfunction and memory decline.

### Mitochondrial dysfunction abnormalities in AlCl3 exposed aging mouse: assessment of related biomarkers

**FIGURE 2:**
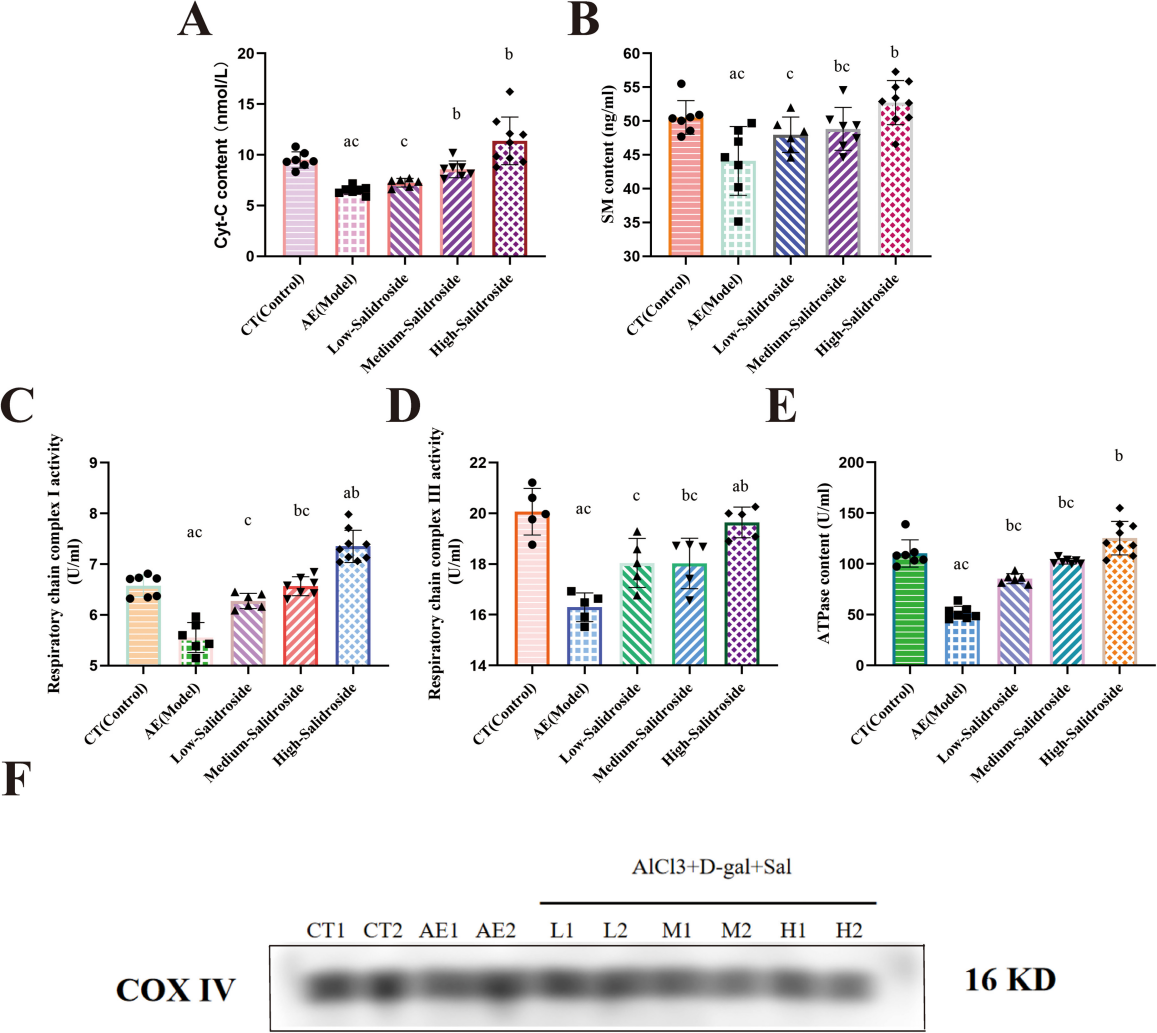
Mitochondrial biomarkers measured in mitochondrial samples isolated from mouse brain neurons. **(A)** Mitochondrial Cyt-C content: the AlCl3 exposed aging mouse showed a significant decrease in mitochondrial Cyt-C levels compared to controls (*P* < 0.05). Salidroside treatment restored Cyt-C levels in a dose-dependent manner, with the high-dose group approaching normal levels. **(B)** SM content: AlCl3 exposed aging mouse had significantly lower SM levels than controls (*P* < 0.05). Salidroside increased SM levels, with the most pronounced effect observed in the high-dose group. **(C)** Mitochondrial respiratory complex I activity: complex I activity was significantly reduced in AlCl3 exposed aging mouse (*P* < 0.05). Salidroside significantly enhanced complex I activity, with the strongest effect in the high-dose group. **(D)** Mitochondrial respiratory complex III activity: Complex III activity was significantly decreased in AlCl3 exposed aging mouse compared to controls (*P* < 0.05). Salidroside restored complex III activity, with high-dose treatment bringing levels close to those of controls. **(E)** ATPase activity: ATPase activity was significantly lower in AlCl3 exposed aging mouse (*P* < 0.05). Salidroside treatment dose-dependently increased ATPase activity. **(F)** Assessment of mitochondrial purity by Western blot analysis: mitochondrial fractions were isolated and subjected to Western blot. COX IV was used as a specific mitochondrial marker to evaluate the purity of mitochondrial preparations. Data are presented as mean ± SD. Statistical significance was determined by one-way ANOVA followed by Tukey’s *post-hoc* test. a:*P* < 0.05 vs. control; b:*P* < 0.05 vs. AE group; c:*P* < 0.05 vs. high-salidroside group.

Following the observation of significant neurotransmitter abnormalities in the aluminum and D-galactose co-exposed mouse model, we further investigated mitochondrial dysfunction in the brain tissue and its relationship with neurotransmitter imbalance. On one hand, mitochondrial respiratory function was impaired at multiple levels, evidenced by markedly decreased activities of respiratory chain complexes I (*F*_(4, 30)_ = 49.65; *P* < 0.0001) and III (*F*_(4, 19)_ = 15.41; *P* < 0.0001), as well as reduced ATPase (*F*_(4, 31)_ = 50.04; *P* < 0.0001) content, indicating disrupted energy metabolism (Figures 2C–E). On the other hand, mitochondrial membrane integrity was compromised, as reflected by a significant reduction in cytochrome c (Cyt-C) (*F*_(4, 31)_ = 16.47; *P* = 0.0017) levels (Figure 2A). These functional impairments may underlie the pathological basis of neurotransmitter metabolic disturbances.

Simultaneously, a significant decrease in sphingomyelin (SM), a key mitochondrial membrane lipid component, was observed (Figure 2B). SM plays a critical role in maintaining mitochondrial membrane fluidity and stability; its depletion likely exacerbates membrane structural disruption, leading to respiratory chain dysfunction and abnormal protein aggregation, thereby further affecting normal neurotransmitter metabolism and signaling. Treatment with Salidroside (Sal) dose-dependently restored SM content (*F*_(4, 31)_ = 6.552; *P* = 0.0002), Cyt-C levels (*F*_(4, 31)_ = 16.47; *P* < 0.0001), and respiratory chain complex [activitiescomplexes I (*F*_(4, 30)_ = 49.65; *P* < 0.0001) and III (*F*_(4, 19)_ = 15.41; *P* < 0.0001)], thereby improving mitochondrial membrane function and energy metabolism (Figures 2A–E). These findings reveal a close association between neurotransmitter abnormalities and mitochondrial membrane lipid remodeling, highlighting the protective role of SM in maintaining mitochondrial function and neuronal homeostasis. Aluminum and D-galactose co-exposure not only induces neurotransmitter imbalance but also causes significant mitochondrial dysfunction by disrupting mitochondrial membrane lipid composition, particularly SM. Salidroside alleviates these damages through modulating sphingolipid metabolism and preserving membrane integrity, demonstrating its potential therapeutic value. In order to test the effect of mitochondrial extraction, the detection of mitochondrial reference protein was performed (Figure 2F).

### Investigation of abnormal protein accumulation and APP processing following mitochondrial dysfunction: SAL treatment in like dementia model

**FIGURE 3:**
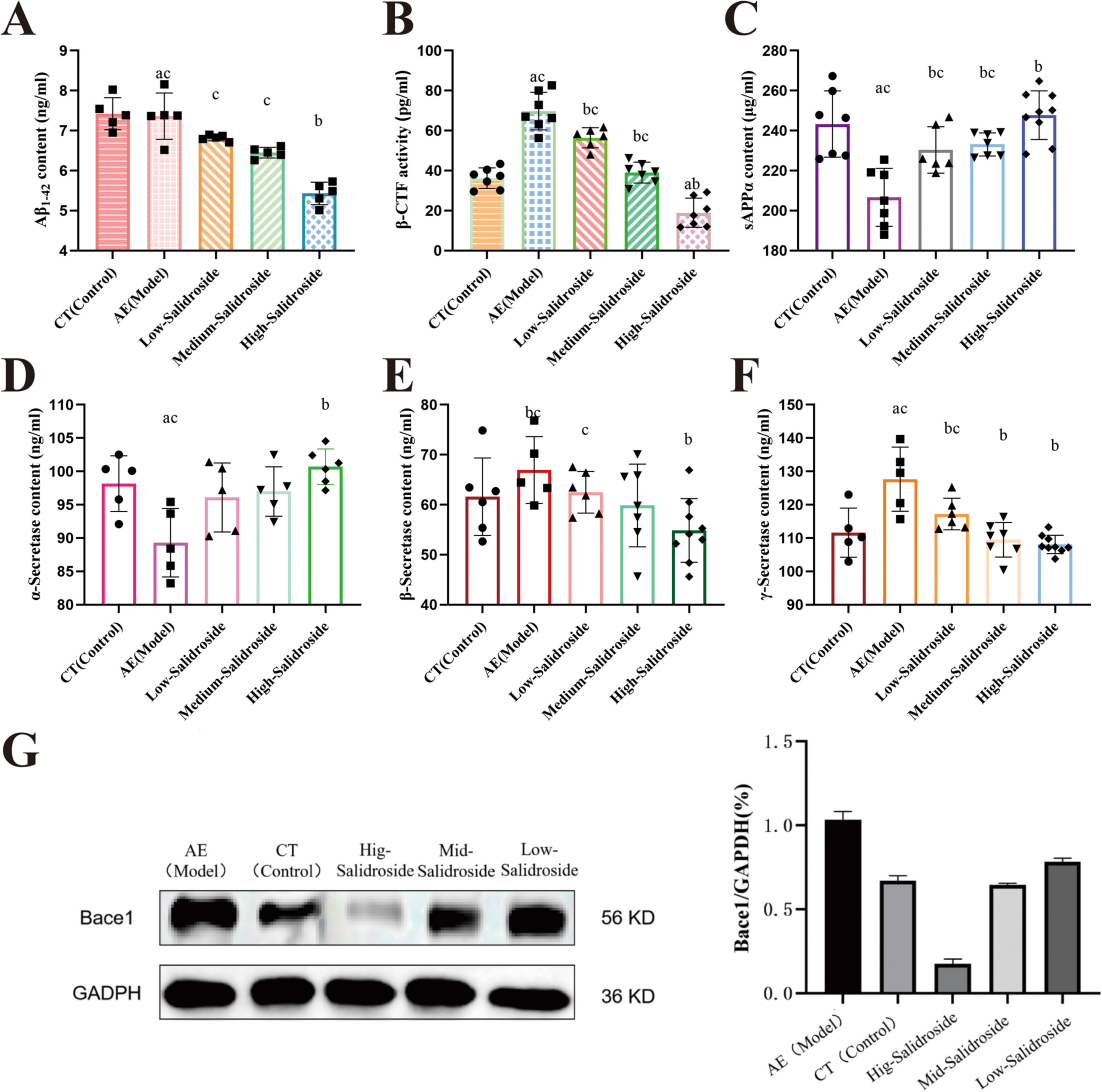
Quantification of Aβ1-42 and β-CTF in mitochondrial samples, and sAPPα, α-/β-/γ-Secretase, and Bace1 in mouse brain homogenates. **(A)** Levels of Aβ1-42. AlCl3 exposed aging mouse showed a significant increase in mitochondrial Aβ1-42 compared to controls (*P* < 0.05). Salidroside administration decreased Aβ1-42 levels in a dose-dependent manner, with high-dose treatment restoring levels close to those of the control group. **(B)** Levels of β-CTF. β-CTF levels were significantly elevated in AlCl3 exposed aging mouse compared to controls (*P* < 0.05). Salidroside treatment reduced β-CTF accumulation, especially in the high-dose group. **(C)** Levels of sAPPα. Compared with the control group, sAPPα levels were significantly decreased in AlCl3 exposed aging mouse (*P* < 0.05). Salidroside treatment restored sAPPα levels in a dose-dependent manner, with the high dose group approaching baseline levels. **(D)** Levels of α-secretase. The content of α-secretase was significantly reduced in the AlCl3 exposed aging mouse (*P* < 0.05). Treatment with low, medium, and high doses of salidroside dose-dependently restored α-secretase levels. **(E)** Levels of β-secretase. β-secretase levels were significantly elevated in the AlCl3 exposed aging mouse compared to controls (*P* < 0.05). Salidroside treatment gradually reduced β-secretase levels, with the high dose group approaching control levels. **(F)** Levels of γ-secretase. γ-secretase levels were significantly increased in the AlCl3 exposed aging mouse (*P* < 0.05). Salidroside administration dose-dependently decreased γ-secretase levels, with the strongest effect observed in the high dose group. **(G)** Western blot analysis of Bace1 expression. Bace1 expression was significantly increased in AlCl3 exposed aging mouse, while salidroside treatment inhibited Bace1 expression. GAPDH was used as the internal control. Data are presented as mean ± SD. Statistical significance was determined by one-way ANOVA followed by Tukey’s *post-hoc* test. a:*P* < 0.05 vs. control; b:*P* < 0.05 vs. AE group; c:*P* < 0.05 vs. high-salidroside group.

In the model group subjected to combined aluminum and D-galactose exposure, some degree of mitochondrial dysfunction was observed, along with accumulation of neurotoxic proteins Aβ1-42 (*F*_(4, 31)_ = 37.37; *P* < 0.0001) and β-CTF (*F*_(4, 29)_ = 59.34; *P* < 0.0001) within mitochondria (Figures 3A,B, mitochondrial markers). Neuroprotective sAPPα levels (*F*_(4, 31)_ = 11.87; *P* < 0.0001) in brain homogenate showed a decreasing trend (Figure 3C brain homogenate marker), suggesting potential disruption of protein homeostasis. APP undergoes three major cleavage pathways: the α-secretase pathway cleaves APP to produce the neuroprotective soluble sAPPα; the β-secretase (BACE1) pathway generates β-CTF; which is further cleaved by γ-secretase to release the neurotoxic Aβ1-42 peptide. The balance among these pathways is critical for maintaining cellular protein homeostasis and neuronal function. Mitochondrial dysfunction is often accompanied by abnormalities in energy and protein metabolism, and accumulation of abnormal proteins may negatively impact mitochondria and neurons, potentially promoting disease progression.

Further analysis of APP cleavage enzymes indicated a decrease in α-secretase activity (*F*_(4, 21)_ = 5.356; *P* = 0.0238) (Figure 3D brain homogenate marker) and trends toward increased β- (*F*_(4, 23)_ = 7.744; *P* = 0.0256) (Figure 3E brain homogenate marker) and γ-secretase activities (*F*_(4, 27)_ = 10.77; *P* = 0.0016) (Figure 3F brain homogenate marker) in brain homogenate. Western blot results showed a tendency for elevated BACE1 protein expression (Figure 3G brain homogenate marker). These changes possibly contribute to increased cleavage of APP into toxic β-CTF and Aβ1-42 fragments, facilitating their accumulation in cytosol and mitochondria. Salidroside treatment appeared to partially restore α-secretase activity(*F*_(4, 21)_ = 5.356; *P* = 0.0018), inhibit β-(*F*_(4, 23)_ = 7.744; *P* = 0.0003) and γ-secretase activities (*F*_(4, 27)_ = 10.77; *P* < 0.0001), and reduce BACE1 expression, thereby promoting a relative balance in APP processing pathways, alleviating toxic protein accumulation, and improving mitochondrial function.

### Network pharmacology reveals potential mechanisms of salidroside in Alzheimer’s disease

**FIGURE 4:**
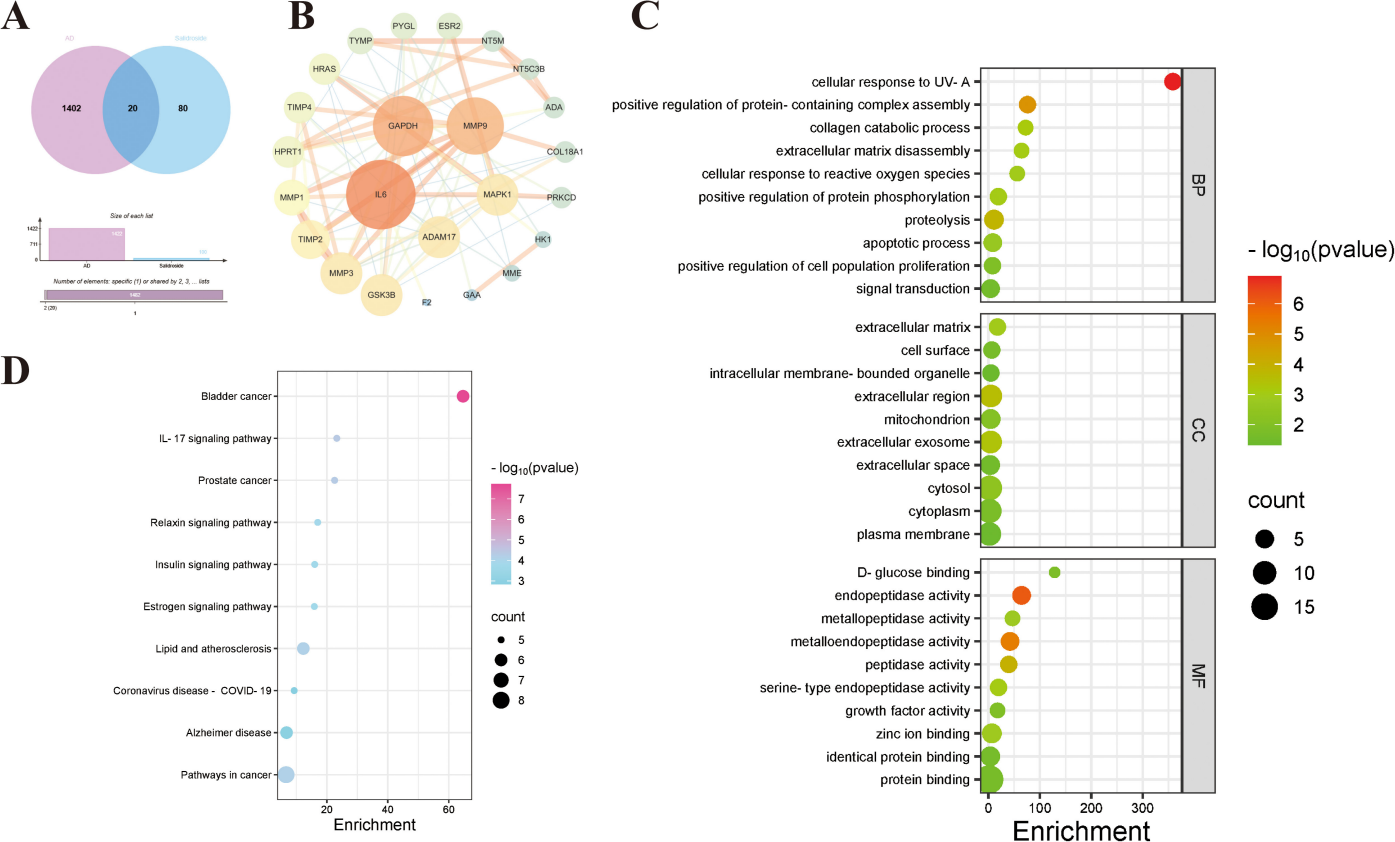
Network and enrichment analyses of putative salidroside targets. **(A,B)** PPI network showing the relationships among putative salidroside targets. Nodes represent proteins and edges indicate interactions; yellow nodes denote key targets, while green nodes represent associated targets. **(C)** GO enrichment analysis of salidroside targets, categorized into BP, CC and MF. The *x*-axis indicates enrichment significance, the *y*-axis lists GO terms, circle size corresponds to the number of genes, and color reflects –log10(*P*-value). **(D)** KEGG pathway enrichment analysis illustrating the distribution of salidroside targets across signaling pathways. The *x*-axis indicates enrichment significance, the *y*-axis shows pathway terms, with circle size representing gene counts and color denoting –log10(*P*-value).

Although the direct role of aluminum in this study remains to be fully clarified, existing evidence suggests that aluminum exposure may exacerbate Alzheimer’s disease pathology by promoting neuroinflammation, oxidative stress, and mitochondrial dysfunction. Based on this premise, network pharmacology was applied to identify potential molecular targets of salidroside (Sal) by integrating its targets with AD-associated genes, yielding 20 candidate overlapping targets (Figure 4A). Protein-protein interaction (PPI) network analysis spotlighted key nodes such as IL6, GAPDH, and MAPK1, which may be implicated in neuroinflammation and apoptotic processes (Figure 4B), consistent with observed neuroprotective and anti-inflammatory effects.

Gene Ontology (GO) enrichment highlighted involvement of these targets in oxidative stress response and proteolysis, predominantly localized to mitochondria and extracellular exosomes, suggesting that salidroside may exert protective effects by regulating mitochondrial function and protein metabolism. Molecular function enrichment in endopeptidase activity and protein binding provides molecular evidence for modulating APP metabolism.

Kyoto Encyclopedia of Genes and Genomes pathway analysis identified significant enrichment of the Alzheimer’s disease pathway, underscoring the central role of APP abnormal cleavage in AD pathogenesis (Figures 4C,D). This result aligns closely with our experimental findings where salidroside improved APP processing and attenuated mitochondrial oxidative stress, supporting the rationale of this study. Additionally, enrichment of the IL-17 signaling pathway implies potential regulation by salidroside of neuroinflammation and related apoptotic mechanisms, offering direction for subsequent investigations into its roles in oxidative stress and apoptosis.

### Antioxidant and anti-apoptotic effects of salidroside in HT-22 cells under OA-induced injury guided by network pharmacology analysis

Building on network pharmacology insights, this study employed an OA-induced injury model in HT-22 cells to investigate the modulatory effects of salidroside on neuronal function.

**FIGURE 5:**
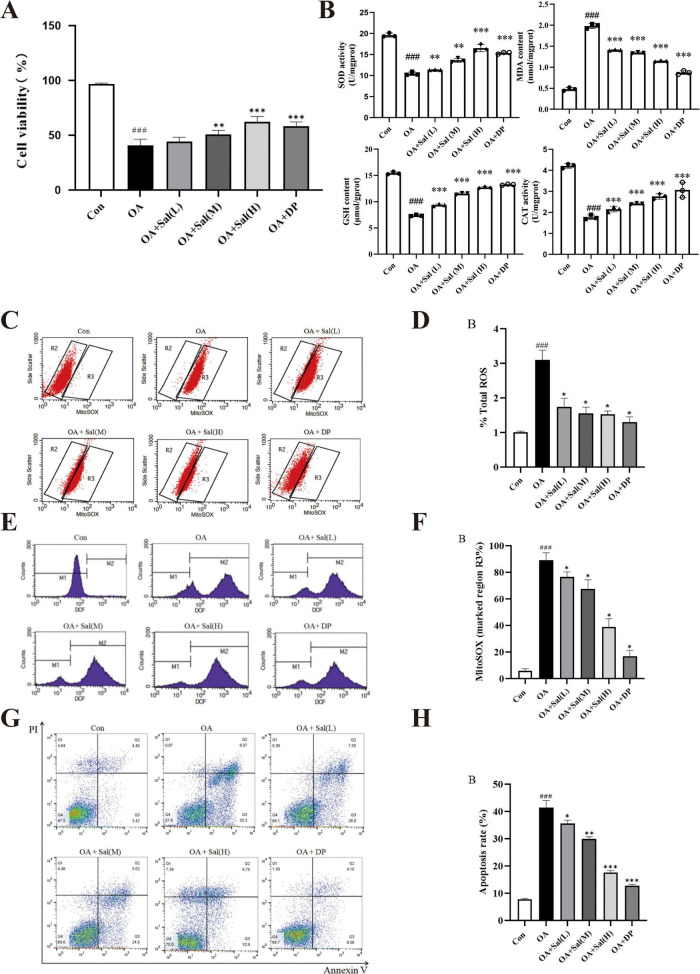
Effects of salidroside on cell viability, oxidative stress, and apoptosis in OX-induced HT-22 cells. **(A)** CCK-8 assay showing that OX treatment markedly reduced cell viability compared with control (Con). Salidroside at low, medium, and high doses significantly rescued viability in a dose-dependent manner, comparable to the positive control drug DP. **(B)** Antioxidant defense and lipid peroxidation markers: SOD activity was significantly decreased by OX and restored by salidroside in a dose-dependent manner. Malondialdehyde (MDA) levels were elevated in the OX group, indicating increased lipid peroxidation. Salidroside dose-dependently decreased MDA, with the high-dose group nearly normalized to control levels. Glutathione (GSH) content was significantly reduced by OX and restored by salidroside in a dose-dependent manner. Catalase (CAT) activity was suppressed by OX and markedly enhanced upon salidroside treatment, most prominently in the high-dose group. **(C,D)** Total reactive oxygen species (ROS) levels measured by flow cytometry showed a robust increase in intracellular ROS in OX-treated cells (M2 region). Salidroside dose-dependently attenuated ROS accumulation, as quantified in bar graphs. **(E,F)** Mitochondrial ROS (mtROS) levels were significantly elevated in OX cells (R3 region), whereas salidroside suppressed mtROS in a dose-dependent manner, with the high-dose group approaching basal levels. **(G,H)** Apoptosis analysis indicated that OX treatment significantly increased early and late apoptotic cell populations. Salidroside reduced apoptotic rates dose-dependently, with the high-dose group showing apoptosis levels close to controls. All experiments were conducted using HT-22 cells. Data are presented as mean ± SD (*n* = 3). Statistical analysis was performed using one-way ANOVA followed by Tukey’s *post-hoc* test. ^###^*P* < 0.001 vs. control group; *P < 0.05, **P < 0.01, ***P < 0.001 vs. OX group.

Cell viability assays indicated a notable reduction in survival following OA exposure compared to controls, suggesting impaired cellular function due to oxidative insult. Salidroside treatment at low, medium, and high doses exerted varying degrees of protective effects, with a gradual improvement in viability noted, especially significant at the highest dose, where recovery was comparable to the positive control DP (*P* < 0.01; Figure 5A).

Regarding antioxidant defense, OA exposure caused a considerable increase in lipid peroxidation marker MDA, while intracellular antioxidants such as GSH and catalase (CAT) activity decreased. Salidroside administration dose-dependently reduced MDA levels; the high-dose group showed values approaching those of controls. Concurrently, GSH content and CAT activity displayed partial restoration (*P* < 0.01; Figure 5B), indicative of redox homeostasis modulation.

Flow cytometric analysis revealed that OA markedly elevated total intracellular ROS levels (*P* < 0.01; Figures 5C,D), whereas salidroside treatment showed a tendency to suppress ROS accumulation in a dose-responsive manner. Similar trends were observed with mitochondrial ROS (mtROS), where salidroside reduced OA-induced mtROS increases, tending toward baseline levels at higher doses (*P* < 0.01; Figures 5E,F).

Apoptotic analysis demonstrated raised proportions of early and late apoptotic cells following OA stimulation. Salidroside treatment was associated with decreasing apoptosis rates, again in a dose-dependent fashion, with high-dose groups nearing control values (*P* < 0.01; Figures 5G,H). Furthermore, mitochondrial function assays showed that OA reduced activities of respiratory chain complexes I, III, and ATPase compared to controls, indicating impaired energy metabolism. Salidroside treatment improved these activities, with increasing doses correlating to greater functional recovery (Table 7).

**TABLE 7:** Determination of mitochondrial function in HT-22 cells ($\bar{x}$± s).

Group	Activities of respiratory chain complexes I (U/mgprot)	Activities of respiratory chain complexes III (U/mgprot)	ATPase activity (μmol/gprot)
Con	8.15 ± 2.07	10.26 ± 2.17	196.15 ± 21.54
OA	2.70 ± 0.59^#^	1.79 ± 0.34^#^	71.13 ± 9.26^#^
OA+Sal(L)	5.02 ± 1.38*	4.91 ± 1.08*	90.16 ± 10.44*
OA+Sal(M)	5.68 ± 1.82*	6.07 ± 1.35*	121.78 ± 14.64*
OA+Sal(H)	6.14 ± 1.61*	7.52 ± 1.64*	158.17 ± 18.25*
OA+DP	6.39 ± 1.77*	7.92 ± 1.48*	164.83 ± 17.61*

Measurement results of mitochondrial function in HT-22 cells. Data met normality and homogeneity assumptions. One-way ANOVA showed significant differences between the model and control groups, and between treatment and model groups. (^#^*p*<0.05) vs. control; (*p<0.05) vs. OA.

Taken together, these findings suggest that salidroside may attenuate OA-induced oxidative stress and dysfunction by enhancing antioxidant defenses, lowering ROS burden, reducing apoptosis, and restoring mitochondrial respiratory chain function, thereby supporting neuronal survival. While further mechanistic elucidation is warranted, these results highlight the potential neuroprotective effect of salidroside.

### Protective effects of salidroside on mitochondrial protein aggregation: co-localization of CTF and mitochondria in Aβ1-42-treated HT-22 cells

Immunofluorescence co-localization analysis was performed using confocal microscopy to assess the spatial relationship between endogenous C-terminal fragments (CTF) and mitochondria in HT-22 cells. Mitochondria were labeled with Mito-tracker CMXRos (red fluorescence), endogenous CTF was detected by FITC-conjugated secondary antibodies (green fluorescence), and nuclei were stained with DAPI (blue).

In control cells, green fluorescence corresponding to endogenous CTF was barely detectable, indicating low expression levels. Mitochondria exhibited typical elongated tubular morphology with limited co-localization between CTF and mitochondria. Following treatment with human Aβ1-42, green fluorescence intensity increased markedly, with predominant localization overlapping or adjacent to mitochondrial signals, suggesting enhanced aggregation of endogenous CTF around mitochondria. Concurrently, cell morphology exhibited changes characterized by a more rounded appearance.

**FIGURE 6:**
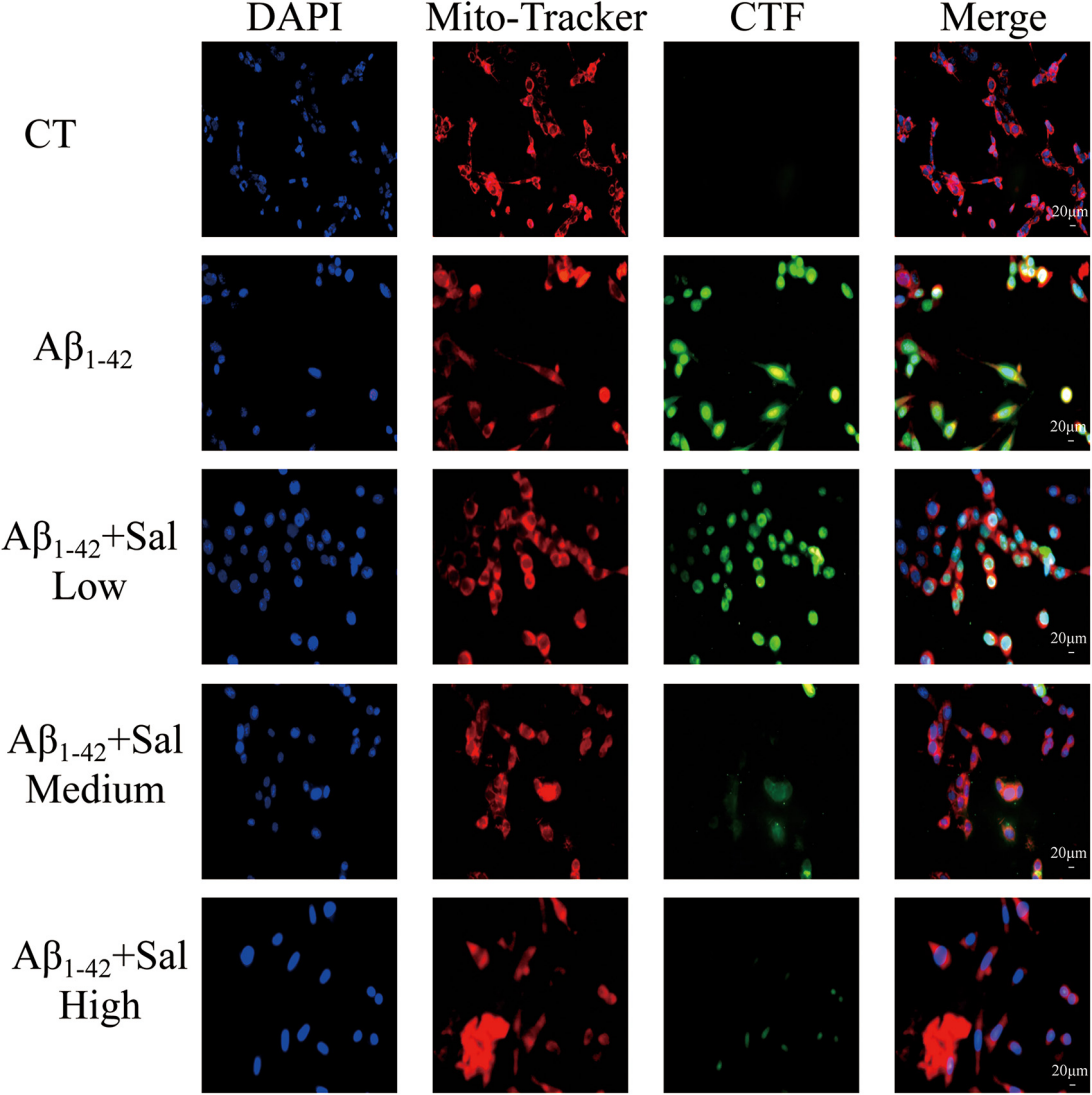
Protective effects of salidroside on mitochondrial protein aggregation: co-localization of CTF and mitochondria in Aβ1-42-treated HT-22 cells. Immunofluorescence co-localization of mitochondria and CTF in HT-22 cells. Mitochondria were labeled with Mito-tracker CMXRos (red), endogenous C-terminal fragments were detected using a FITC-conjugated secondary antibody (green), and nuclei were stained with DAPI (blue). Images were captured using a fluorescence microscope.

After intervention with salidroside, green fluorescence intensity was reduced compared to the Aβ1-42-only group, indicating a decrease in the degree of CTF aggregation. Mitochondrial morphology showed partial recovery towards an elongated shape, and alterations in cell shape appeared less pronounced. These observations imply that salidroside may modulate Aβ1-42-induced endogenous CTF aggregation on mitochondria (Figure 6).

## Discussion

Brain aging encompasses both physiological and pathological processes, with the latter strongly implicated in the onset of neurodegenerative disorders such as Alzheimer’s disease. In the early stages, physiological brain aging and AD share overlapping clinical manifestations, pathological hallmarks, biochemical alterations, and underlying molecular mechanisms, particularly involving abnormal protein aggregation. This convergence suggests that brain aging may represent a prodromal or predisposing phase of neurodegenerative pathology, with age-related pathological changes potentially accelerating the development and progression of diseases such as AD (Mattson and Arumugam, 2018).

Among environmental factors implicated in AD pathogenesis, aluminum (Al) neurotoxicity has garnered increasing attention. Elevated aluminum concentrations have been detected in the brains of AD patients, where aluminum is postulated to exacerbate classical pathological features such as β-amyloid plaque aggregation and neurofibrillary tangle formation. Aluminum exposure induces oxidative stress and neuroinflammation, both of which contribute to neuronal injury and cognitive decline. Additionally, aluminum disrupts metal homeostasis–especially iron regulation–promoting ROS generation and amplifying oxidative damage within neural tissue. Although a definitive causal relationship remains to be conclusively established, converging epidemiological and experimental evidence supports the notion that brain aluminum accumulation acts as an environmental risk factor that can accelerate or worsen AD pathology. Other heavy metals, including cadmium and lead, also contribute to neurodegenerative processes through oxidative damage and interference with neurotransmitter systems (Zawia and Basha, 2005). In this context, combined aluminum and D-galactose administration offers a relevant and widely accepted animal model to mimic aging-related cognitive impairment linked to cumulative oxidative and inflammatory brain injury, providing a suitable platform to evaluate neuroprotective interventions.

Brain aging is characterized histologically by neuronal loss and reactive gliosis. Compared with cognitively healthy elderly individuals, AD patients exhibit greater neuronal and synaptic loss, correlating with regional brain atrophy (Terry et al., 1991). Senile plaques and neurofibrillary tangles remain hallmark pathological lesions of AD, with their density positively correlating with dementia severity (Braak and Braak, 1991). Notably, these inclusions are also discernible in normally aging brains, especially beyond the eighth decade of life, resulting in no statistically significant difference between healthy elderly and AD patients in terms of plaque and tangle burden (Jellinger, 2020). Consequently, while senile plaques and neurofibrillary tangles retain diagnostic utility for early-onset AD, their specificity diminishes markedly in the oldest old.

Our present study provides evidence that salidroside, a phenolic glycoside derived from *Rhodiola rosea*, exerts multifaceted neuroprotective effects in AD models. Epidemiological forecasts predict a substantial increase in dementia prevalence worldwide over the coming decades, underscoring the urgency for effective therapeutic options (Zhang et al., 2020). AD is a multifactorial neurodegenerative disorder characterized by extracellular amyloid beta (Aβ) plaque deposition, tau hyperphosphorylation, mitochondrial dysfunction, oxidative stress, and progressive neuronal loss (Du et al., 2022; Lee et al., 2013).

Specific binding of cholesterol to C99 is a sensitive function of the pH encountered *in vivo*, with key E22 and D23 residues serving as a “pH switch” controlling C99-cholesterol binding (Panahi et al., 2016). Elevated levels of C99 (the β-secretase cleavage product of APP) promote the remodeling of mitochondria-associated endoplasmic reticulum membranes (MAMs) by promoting cholesterol clustering, which in turn activates ACSL4 and alters the composition of phosphatidylcholine (PC). This mechanism has been validated in various Alzheimer’s disease (AD) models, as well as in fibroblasts, neurons, and immune cells derived from both familial and sporadic AD patients, manifesting as persistently elevated C99 levels, increased ACSL4 activity, and enrichment of PC species containing polyunsaturated fatty acids, ultimately leading to lipid imbalance and membrane dysfunction. This study reveals the critical role of MAMs as dynamic lipid-regulatory hubs in coordinating ACSL4-dependent membrane remodeling and highlights that MAM dysfunction is a key mechanism underlying lipid abnormalities in AD (Montesinos et al., 2025). C99, the C-terminal processing product of the amyloid precursor protein (APP) derived from its cleavage by β-secretase, is present in MAM, that its level is increased in AD, and that this increase reduces mitochondrial respiration, likely via a C99-induced alteration in cellular sphingolipid homeostasis (Area-Gomez et al., 2018). In the early pathological process of Alzheimer’s disease, APP C-terminal fragments (CTFs) promote abnormal cholesterol accumulation in lysosomes by localizing to late endosome/lysosome–endoplasmic reticulum contact sites and reducing ER-to-lysosome calcium refilling. This cholesterol buildup driven by excessive CTFs is a key step that leads to the progressive collapse of the endolysosomal system (Bretou et al., 2024). The accumulation of APP-CTFs, independently of Aβ, directly triggers mitochondrial morphological abnormalities, increased reactive oxygen species, and impairment of basal mitophagy. Given that defective mitochondrial homeostasis plays a pivotal role in the pathogenesis of Alzheimer’s disease (AD), targeting mitochondrial dysfunction and/or mitophagy through early intervention against APP-CTFs accumulation may represent a relevant therapeutic strategy for AD (Vaillant-Beuchot et al., 2021). Based on the above, we aim to conduct a preliminary investigation into the protective effects of salidroside on mitochondrial dysfunction in early aging, with a specific focus on the involvement of C99-mediated mitochondrial sphingolipid metabolism. Given that the accumulation of APP-CTFs (including C99) independently triggers mitochondrial abnormalities and impairs mitophagy–processes central to Alzheimer’s disease pathogenesis–this study will explore whether salidroside ameliorates early mitochondrial dysfunction by modulating C99-associated sphingolipid signaling at the mitochondria.

A salient mechanism underlying salidroside’s action is its modulation of APP proteolytic processing. Aberrant cleavage of APP by β- and γ-secretases generates toxic β-C-terminal fragments (β-CTF) and Aβ peptides, which aggregate to form amyloid plaques and mediate neurotoxicity. Our data show that salidroside attenuates the activity of these amyloidogenic secretases, thus favoring the non-amyloidogenic α-secretase pathway and elevating levels of the neuroprotective soluble APP alpha fragment (sAPPα). This shift not only reduces Aβ burden but also mitigates mitochondrial dysfunction, oxidative stress, and apoptotic signaling, thereby preserving neuronal integrity and function. Such multi-level regulation of APP processing provides a promising advantage over therapeutics targeting a single pathological pathway (Zhang P. et al., 2023).

Furthermore, salidroside ameliorated sphingomyelin (SM) metabolism, a critical component of neuronal membranes involved in signal transduction, membrane fluidity, and intercellular communication. AD-related secretase dysregulation and oxidative injury disrupt SM homeostasis, leading to membrane abnormalities and impaired cellular functionality. Our results indicate that salidroside restores SM levels by modulating APP processing and associated signaling, decreasing lipid peroxidation and enhancing membrane stability and fluidity. These effects likely preserve membrane-associated receptor function and neuronal resilience against oxidative insults, thereby supporting synaptic plasticity. Regulation of SM metabolism thus emerges as an integral aspect of salidroside’s neuroprotective profile.

Mitochondrial function assays revealed that salidroside enhanced respiratory complex I and III activities, improved ATP synthase function, and preserved cytochrome c retention, while concomitantly reducing ROS generation and lipid peroxidation. Simultaneously, activities of endogenous antioxidant enzymes–including superoxide dismutase (SOD), catalase (CAT), and glutathione (GSH)–were significantly upregulated. These findings extend previously reported antioxidant and anti-apoptotic properties of salidroside, reinforcing its role as a multitarget modulator of AD-associated pathology (Klemmensen et al., 2024; Wang W. et al., 2020). In contrast to past monotherapies targeting cholinesterase, NMDA receptors, or Aβ alone–which have generally yielded limited clinical success and safety concerns (Cai et al., 2022; Zhang et al., 2016) –salidroside’s pleiotropic effects on inflammation, oxidative stress, and aging-related pathways warrant further mechanistic exploration in AD models (Chen et al., 2020).

Recent *in vitro* studies suggested that salidroside reduces Aβ-induced apoptosis through activation of ERK/Akt signaling (Liao et al., 2019). However, its *in vivo* impact on secretase activity and mitochondrial homeostasis remained elusive. To clarify this, we integrated network pharmacology with both *in vitro* and *in vivo* experiments. Protein-protein interaction network analysis identified key regulatory nodes including IL6, MAPK1, and GAPDH, indicating convergence of neuroinflammatory, oxidative stress, and apoptotic pathways on APP metabolism and mitochondrial function (Xie et al., 2024; Zhang et al., 2024). Biochemical assays subsequently confirmed that salidroside promotes α-secretase activity and increases sAPPα while suppressing β- and γ-secretase activities, thereby reducing β-CTF and Aβ1–42 accumulation. Functional assessments showed restoration of mitochondrial complex I/III activities, ATPase function, and cytochrome c retention, coupled with decreased ROS and lipid peroxidation, alongside enhanced antioxidant defenses. These findings align with emerging literature implicating secretase imbalance in mitochondrial APP accumulation, synaptic dysfunction, and cognitive deficits in AD transgenic models (Butterfield and Boyd-Kimball, 2018).

**FIGURE 7:**
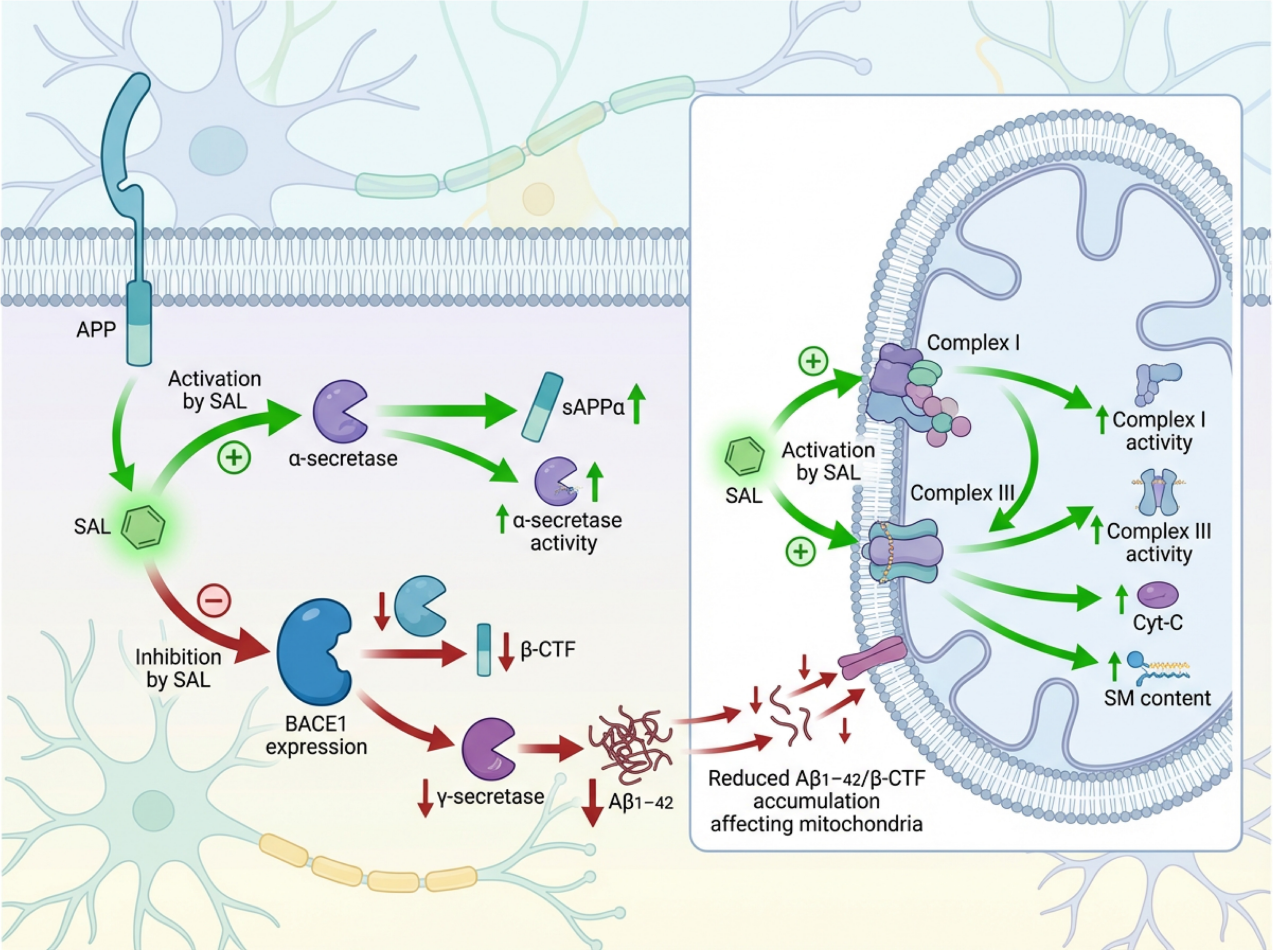
Mechanistic diagram of salidroside’s effects on mitochondrial protein aggregation and mitochondrial function via the APP proteolytic pathway like dementia models.

By normalizing secretase activity and bolstering mitochondrial integrity, salidroside may disrupt this pathogenic feedback loop, offering a more integrated therapeutic strategy than single-target treatments (Figure 7).

Despite these advances, several limitations warrant consideration. The D-galactose/aluminum (D-gal/Al^3 +^) model, while effective for inducing aging-like cognitive deficits, lacks hallmark amyloid plaque and tau pathologies characteristic of human AD. Similarly, HT-22 neuronal cells cannot fully replicate the complexity of *in vivo* neural circuitry. Future research should validate these findings in transgenic AD models and conduct long-term evaluations of salidroside’s safety, pharmacokinetics, and blood–brain barrier permeability. These parameters are critical for clinical translation. Open questions remain regarding whether salidroside’s modulation of secretase activity and mitochondrial function involves upstream regulators such as Nrf2, PI3K/Akt, or sirtuin family proteins. Given AD’s multifactorial etiology, exploring rational combination therapies incorporating salidroside with tau aggregation inhibitors or anti-inflammatory agents may further enhance therapeutic efficacy.

In conclusion, this study indicates that salidroside may exert neuroprotective effects in aging models, potentially through modulation of APP processing and reduction of mitochondrial protein aggregation, as well as attenuation of oxidative stress and apoptosis. Given the limited efficacy observed with some single-target therapies in clinical settings, these findings highlight the potential value of multi-mechanistic approaches in aging research. Additionally, salidroside’s natural origin and generally favorable safety profile support its candidacy for further investigation as a therapeutic agent targeting neurodegenerative diseases.

## Data Availability

The original contributions presented in this study are included in this article/Supplementary material, further inquiries can be directed to the corresponding author.
